# Quinotrierixin inhibits proliferation of human retinal pigment epithelial cells

**Published:** 2013-01-07

**Authors:** Chen Chen, Joshua J. Wang, Jingming Li, Qiang Yu, Sarah X. Zhang

**Affiliations:** 1 State Key Laboratory of Ophthalmology, Zhongshan Ophthalmic Center, Sun Yat-sen University, Guangzhou, China; 2 Department of Medicine, Endocrinology and Diabetes, University of Oklahoma Health Sciences Center, Oklahoma City, OK; 3 Harold Hamm Diabetes Center, University of Oklahoma Health Sciences Center, Oklahoma City, OK; 4 Department of Physiology, University of Oklahoma Health Sciences Center, Oklahoma City, OK; 5 Department of Ophthalmology, University of Oklahoma Health Sciences Center, Oklahoma City, OK

## Abstract

**Purpose:**

To investigate the effect of quinotrierixin, a previously reported inhibitor of X-box binding protein 1 (XBP1), on cell proliferation and viability in human retinal pigment epithelium (RPE) cells.

**Methods:**

Subconfluent human RPE cells (ARPE-19) were exposed to quinotrierixin for 16–24 h. Cell proliferation was determined with 3-(4, 5-dimethylthiazolyl-2)-2,5-diphenyltetrazolium bromide assay, hemocytometer counts, and CyQUANT NF Cell Proliferation Assay. Apoptosis was detected with terminal deoxynucleotidyl transferase-mediated uridine 5′-triphosphate-biotin nick end labeling assay. XBP1 mRNA splicing and expression of endoplasmic reticulum stress response genes were determined in cells exposed to thapsigargin in the presence or absence of quinotrierixin. Overexpression of spliced XBP1 was achieved with adenovirus.

**Results:**

Quinotrierixin reduced RPE cell proliferation in a dose-dependent manner without inducing apoptosis. In cells exposed to thapsigargin, quinotrierixin inhibited XBP1 mRNA splicing and PKR-like endoplasmic reticulum kinase activation, and reduced cellular and nuclear levels of spliced XBP1 and C/EBP homologous protein. Paradoxically, quinotrierixin exacerbated endoplasmic reticulum stress-induced phosphorylation of eIF2α, which in turn led to decreased protein translation. Overexpressing spliced XBP1 partially reversed the inhibition of cell proliferation by quinotrierixin. These results suggest that inhibiting XBP1 splicing contributes to quinotrierixin’s negative effect on RPE cell proliferation, but other mechanisms such as reduction of protein translation are also involved.

**Conclusions:**

Quinotrierixin inhibits RPE cell proliferation and may be used as a novel antiproliferative drug for treating proliferative vitreoretinopathy. Future studies are needed to investigate the in vivo effect of quinotrierixin on RPE proliferation in animal models of proliferative vitreoretinopathy.

## Introduction

Proliferative vitreoretinopathy (PVR), an anomalous retinal scarring process following retinal detachment, is the most common cause of failure in rhegmatogenous retinal detachment surgery [1]. PVR is defined as the growth and contraction of cellular membranes within the vitreous cavity and on both sides of the retinal surfaces [2]. Contraction of the membranes distorts the inner retina and causes redetachment of the retina, resulting in poor vision recovery and ultimately irreversible blindness. Compelling evidence demonstrates that retinal pigment epithelial (RPE) cells play a vital role in the development of contractile membranes [3]. Once released into the vitreous through retinal breaks, RPE cells proliferate and migrate through the vitreous cavity or on the retinal surface, and secrete growth factors and cytokines promoting fibrotic membrane formation. RPE cells also undergo epithelial–mesenchymal transition and transform into fibroblast-like cells, producing excessive collagen and fibronectin that constitute the extracellular matrix of PVR membranes. Furthermore, RPE cells can pull in collagen fibers in a hand-over-hand manner and exert tractional forces, resulting in PVR [4].

Over the past 15 years, significant progress has been made in PVR pharmacotherapy. Troglitazone was reported to dose-dependently inhibit transforming growth factor beta 2 (TGFβ2)-induced collagen type I (COLI) and fibronectin (FN) overexpression in RPE cells, as well as TGFβ2-induced cell migration [5]. Other drugs targeting the TGFβ pathway have also been studied extensively. For example, decorin [6], fasudil [7], and simvastatin [8] all exhibited similar inhibitory effects on aberrant fibrosis of proliferative tissue. Meanwhile, emerging evidence suggests that inhibition of RPE cell proliferation may be a new treatment for PVR. In fully developed healthy eyes, RPE cells normally do not undergo mitosis. However, under pathologic conditions such as retinal detachment or ocular trauma, RPE cells are exposed to serum components and become activated. Proliferation of activated RPE cells is believed to be a central event in the pathogenesis of PVR [9,10]. In recent years, several pharmaceutical inhibitors of RPE cell proliferation have been identified. Retinoids inhibited proliferation of cultured bovine RPE cells, among which all-trans-retinoic acid exhibited the most potent inhibitory effect [11]. Similarly, 5-fluorouracil (5-FU) inhibited contraction of collagen lattices containing RPE cells and proliferation of RPE cells [12]. In addition, hydroxy derivatives of minoxidil [13], vitamin E, and vitamin C [14,15] all exhibited inhibitory effects on RPE cell proliferation. Despite the agents’ potent activity in inhibiting RPE proliferation, the clinical application of these pharmacological agents is limited, largely due to high drug toxicity. New medications with higher safety are desperately needed.

Quinotrierixin is a novel member of the triene-ansamycin group antibiotics. It was originally identified by the Tashiro group in 2007, in an effort to screen for inhibitors of endoplasmic reticulum (ER) stress-induced X-box binding protein 1 (XBP1) mRNA splicing [16]. Isolated from the cultured broth of *Streptomyces* sp. PAE37, quinotrierixin demonstrates chemical characteristics, i.e., possesses NH/OH (3450 cm^−1^), ester (1730 and 1200 cm^−1^), and amide (1640 and 1500 cm^−1^) functionalities, that indicate this agent belongs to the triene-ansamycin family [16]. The molecular formula of quinotrierixin is C_37_H_50_N_2_O_8_S (MW 682), with a SCH3 group speculated at C-21 or C-23 [16]. Quinotrierixin inhibits thapsigargin-induced XBP1 activation in a dose-dependent manner with an IC_50_ of 0.067 μM (mRNA) or 0.082 μM (luciferase) [17]. Quinotrierixin also demonstrates potent inhibitory effect on tumor cell growth [17]. Interestingly, a recent study from the same group shows that quinotrierixin inhibits expression of other unfolded protein response (UPR)-related genes, such as the 78 kDa glucose-regulated protein (GRP78) and C/EBP homologous protein (CHOP), and reduces protein synthesis [18]. However, the effect of quinotrierixin on normal cell proliferation and survival has not been studied. In the present study, we investigated the effect of quinotrierixin on RPE cell proliferation and viability and explored quinotrierixin’s potential mechanism. Our results suggest that quinotrierixin inhibits RPE cell proliferation through, at least in part, inhibiting XBP1 splicing, and, therefore, may be used as an antiproliferative agent for treating PVR.

## Methods

### Cell culture

ARPE-19 cells were purchased from American Type Culture Collection (ATCC, Manassas, VA) and maintained in Dulbecco's Modification of Eagle's Medium (DMEM)/Ham's F-12 50/50 mix medium (Cellgro, Manassas, VA) containing 10% fetal bovine serum (FBS; Gibco, Grand Island, NY) and 1% antibiotic/antimycotic solution (containing 10,000 units/ml penicillin G, 10 mg/ml streptomycin sulfate, and 25 µg/ml amphotericin B; Cellgro). For the proliferation assay, cells were seeded at 30%–50% confluence. For all other experiments, cells were allowed to grow to 100% confluence and quiescence overnight with serum-free DMEM/F12 medium before treatment.

### 3-(4, 5-dimethylthiazolyl-2)-2,5-diphenyltetrazolium bromide assay

MTT (3-(4, 5-dimethylthiazolyl-2)-2,5-diphenyltetrazolium bromide) assay was performed using the MTT Cell Proliferation Assay kit (ATCC) following the manufacturer’s instructions. Briefly, at the end of each treatment, medium was refreshed and 30 μl MTT reagent was added to each well of a 24 well plate. Cells were incubated at 37 °C for 2 h. Then, 300 μl detergent reagent was added to each well, and cells were incubated at room temperature in the dark for 4 h to completely dissolve the precipitation. The absorbance was measured at 570 nm with a microplate reader (Perkin Elmer, Waltham, MA). Cell numbers were calculated according to a standard curve.

### Cell counting

Cells were trypsinized, and viable cells were counted using a hemocytometer. Briefly, 10 μl cell suspension was introduced into one of the V-shaped wells of the hemocytometer, and the area under the coverslip filled by capillary action. The hemocytometer was then placed on the microscope stage, and the cell number was counted under low magnification.

### Cellular DNA content measurement

Cellular DNA content was measured with the CyQUANT NF Cell Proliferation Assay Kit (Life Technologies, Grand Island, NY) as described previously [19]. Briefly, 5×10^3^ ARPE-19 cells were seeded in a 96-well plate for 24 h followed by treatment with 0.5 μM quinotrierixin (QT) for 16 h. Then ARPE-19 cells were incubated with 1× dye binding solution at 37 °C for 30 min in the dark. Fluorescence was detected with a microplate reader (Perkin Elmer) with excitation at 485 nm and emission at 530 nm.

### Terminal deoxynucleotidyl transferase-mediated uridine 5′-triphosphate-biotin nick end labeling assay

Terminal deoxynucleotidyl transferase-mediated uridine 5′-triphosphate-biotin nick end labeling (TUNEL) assay was performed using the In Situ Cell Death Detection Kit, TMR red (Roche Diagnostics Corp., Indianapolis, IN) per the manufacturer’s instruction. Briefly, cells were fixed with 4% paraformaldehyde (PFA) at room temperature for 1 h, followed by permeabilization for 2 min on ice in 0.1% citrate buffer containing 0.1% Triton X-100. Then coverslips were incubated at 37 °C in the TUNEL reaction mix containing nucleotides and terminal deoxynucleotidyl transferase (TdT). After being washed thoroughly, the coverslips were mounted on a slide with a mounting medium containing 4'-6-diamidino-2-phenylindole (DAPI; Vector Laboratories, Burlingame, CA) and observed under a fluorescence microscope.

### Reverse transcription polymerase chain reaction (RT–PCR)

Total RNA was extracted from ARPE-19 cells using the E.Z.N.A. Total RNA Kit I (Omega Bio-Tek, Norcross, GA) following the manufacturer’s instructions. cDNA was synthesized using a Maxima First Strand cDNA Synthesis Kit (Fermentas, Glen Burnie, MD). RT–PCR was performed using the cDNA template and PCR Master Mix (Fermentas) as described previously [20,21]. Each reaction system included 25 µl PCR Master Mix, 22 µl distilled water, 1 µl of the forward primer, 1 µl of the reverse primer and 1 µl of the cDNA template. The primers for human XBP1 were 5′-TTA CGA GAG AAA ACT CAT GGC-3′ and

5′-GGG TCC AAG TTG TCC AGA ATG C-3′. PCR cycle was: 95 °C for 5 min, following by 95 °C for 1 min; 58 °C for 30 s; 72 °C for 30 s with 34 additional repeats, then 72 °C for 5 min. PCR products were resolved on a 2.5% agarose/1× TAE gel. A 289 bp amplicon was generated from unspliced XBP1; a 263 bp amplicon was generated from spliced XBP1 [21].

### Western blot analysis

To extract the total cellular proteins, cells were lysed in RIPA buffer with a protease inhibitor cocktail, phenylmethylsulfonyl fluoride (PMSF), and sodium orthovanadate (Santa Cruz Biotechnology, Santa Cruz, CA). Nuclear proteins were extracted using a Nuclear Extract Kit (Active Motif, Carlsbad, CA). Protein concentration was quantified using a bicinchoninic acid kit (Pierce Biotechnology, Rockford, IL). Twenty-five micrograms of protein were resolved with sodium dodecyl sulfate–PAGE gel and electrotransferred to nitrocellular membranes. After blocking, membranes were blotted overnight at 4 °C with the following primary antibodies: anti-XBP1 (1:500), anti-p-PKR-like endoplasmic reticulum kinase (PERK; 1:1000), anti-CHOP (GADD153, 1:1000; Santa Cruz), anti-p-eIF2a (1:1000; Cell Signaling Technology, Danvers, MA). After being incubated with horseradish peroxidase–conjugated secondary antibodies, the membranes were developed with chemiluminescence substrate (Thermo Fisher Scientific, Rockford, IL) using a BioSpectrum Advanced Imaging System (UVP, Upland, CA). Membranes were reblotted with anti-β-actin (1:5000; Abcam, Cambridge, MA) for loading control. The bands were semiquantified with densitometry using Vision Works LS (UVP, Upland, CA) image acquisition and analysis software.

### Adenoviral transduction in ARPE-19 cells

ARPE-19 cells at 30%–50% confluence were transduced with adenoviruses expressing spliced XBP1 as described previously [22]. Adenovirus expressing LacZ was used as control. Twenty-four hours after transduction, cells were treated with quinotrierixin (0.5 μM) for another 24 h. Cell proliferation was analyzed with MTT assay and cell counting as described above.

### Statistical analysis

The quantitative data are expressed as mean±standard deviation (SD). Statistical analyses were performed using an unpaired Student *t* test when comparing two groups and one-way ANOVA (ANOVA) with Bonferroni’s multiple comparison test for three groups or more. Statistical differences were considered significant at a p value of less than 0.05.

## Results

### Quinotrierixin reduced retinal pigment epithelium cell proliferation

**Figure 1:**
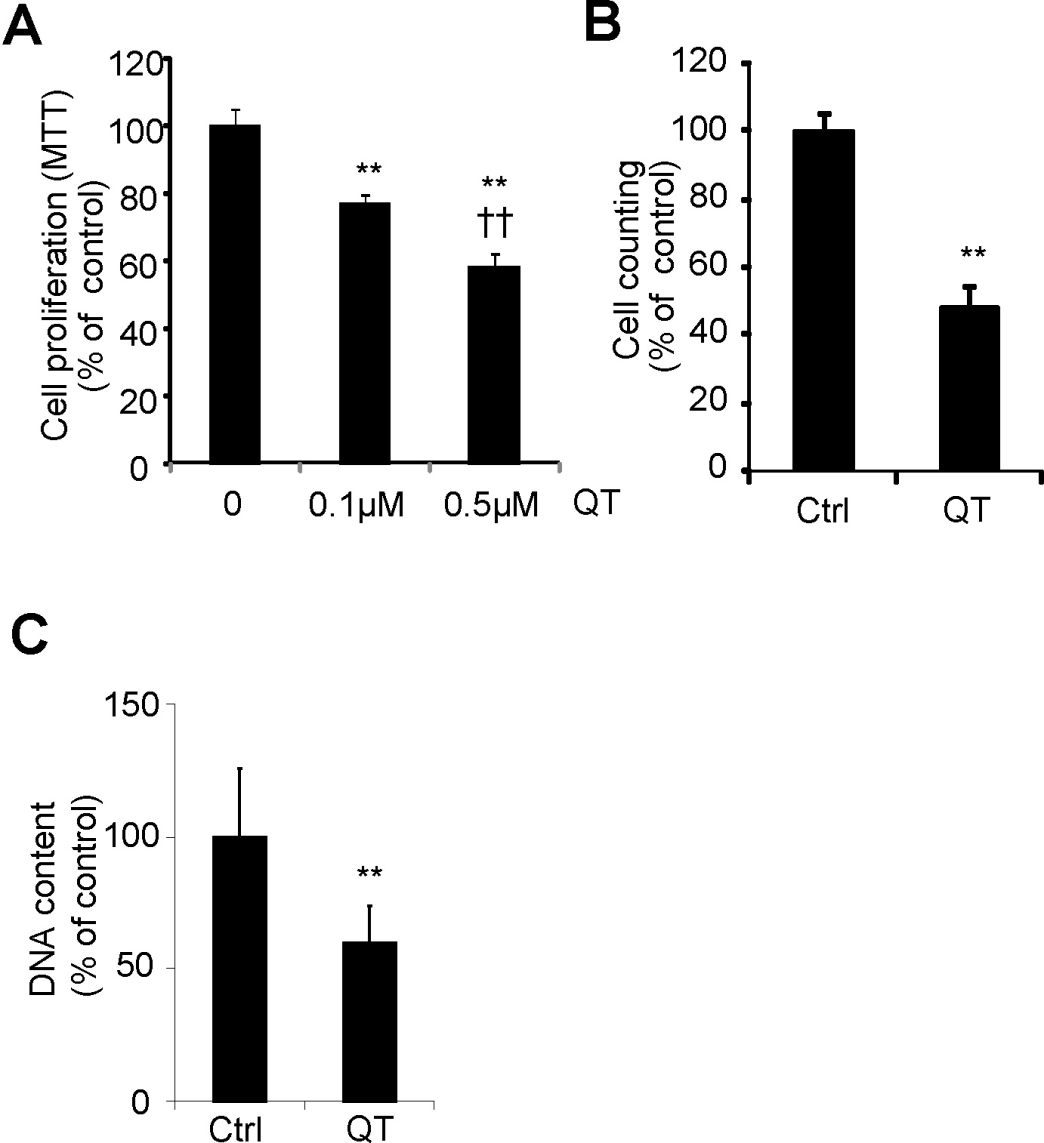
Quinotrierixin suppresses human retinal pigment epithelium cell proliferation. ARPE-19 cells were treated with quinotrierixin (QT), and cell proliferation was determined using 3-(4, 5-dimethylthiazolyl-2)-2,5-diphenyltetrazolium bromide (MTT) assay (n=5; **A**), cell counting (n=3; **B**), or CyQUANT NF Cell Proliferation Assay (n=4, **C**). Quinotrierixin concentration was 0.5 μM unless otherwise indicated. Treatment time was 24 h for panels **A** and **B** and was 16 h for panel **C**. Data are expressed as mean±SD **p<0.01 versus control, ††p<0.01 versus quinotrierixin 0.1 μM.

To determine the effect of quinotrierixin on RPE cell proliferation, ARPE-19 cells were treated with quinotrierixin at different doses for 16 h or 24 h. Cell proliferation was quantified with three widely used methods, including MTT assay, cell counting, and cellular DNA content measurement. The MTT assay measures the mitochondrial metabolic rate and indirectly reflects the viable cell number. Results from the MTT assay are shown in Figure 1A. Quinotrierixin at 0.1 μM decreased the viable cell number to 77% of the control, while that at 0.5 μM decreased the viable cell number to 59% of the control. The results were confirmed with two other assays, demonstrating that quinotrierixin at 0.5 μM reduced cell proliferation to 48% by cell counting, and 59% by DNA content measurement (Figure 1B,C).

### Quinotrierixin did not induce retinal pigment epithelium cell apoptosis

**Figure 2:**
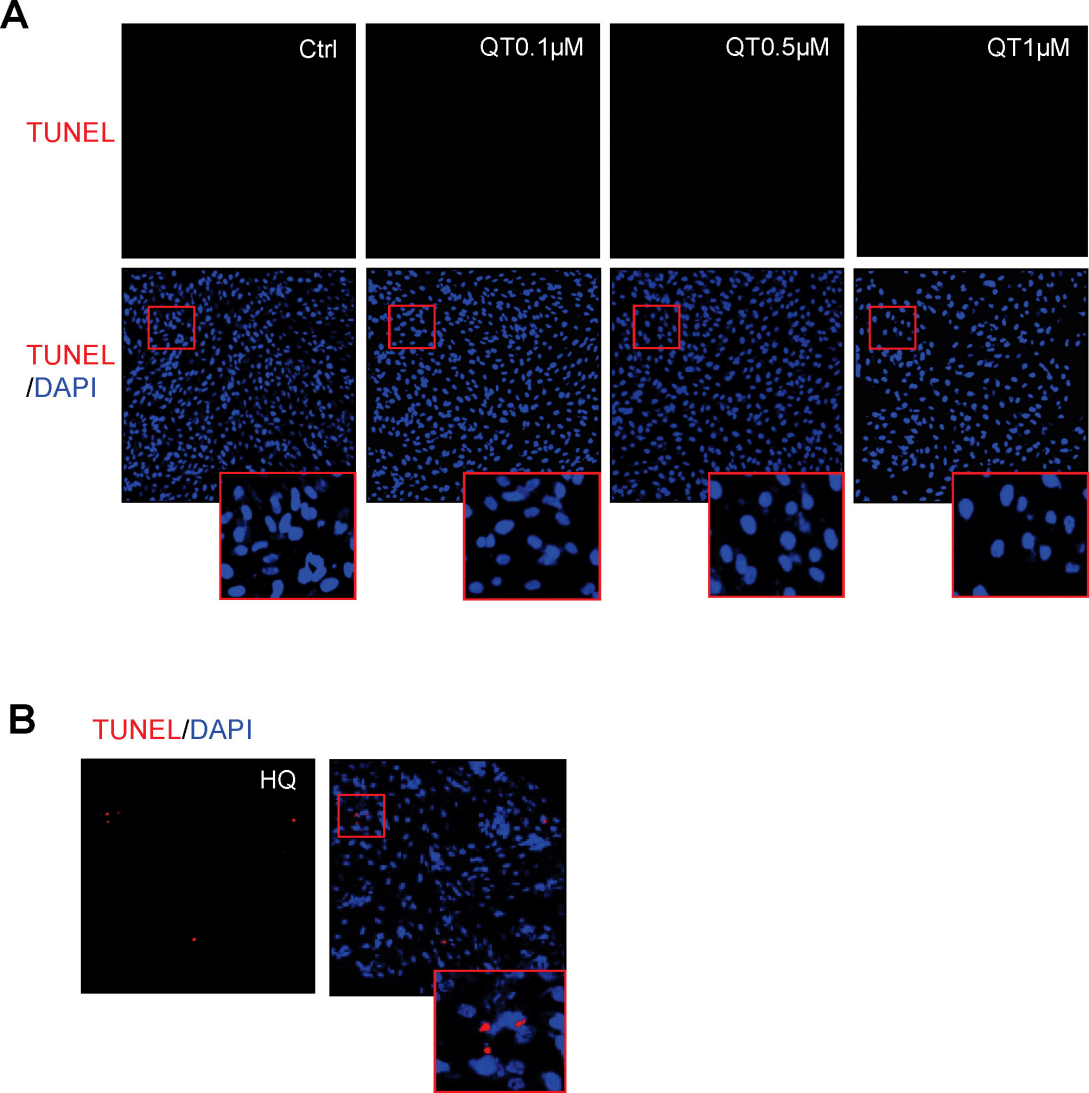
Quinotrierixin does not induce apoptosis in human retinal pigment epithelium cells. **A**: ARPE-19 cells were treated with quinotrierixin 0.1 μM, 0.5 μM, or 1 μM for 24 h. Apoptosis was determined with terminal deoxynucleotidyl transferase-mediated uridine 5′-triphosphate-biotin nick end labeling (TUNEL) assay. **B**: ARPE-19 cells were treated with hydroquinone (HQ) 100 μM as positive control. Red: TUNEL staining of apoptotic cells; Blue: nuclear staining with 4'-6-diamidino-2-phenylindole (DAPI).

To determine whether the inhibitory effect of quinotrierixin on cell proliferation is caused by inducing apoptosis, we examined apoptosis in quinotrierixin-treated APRE-19 cells using TUNEL assay. No apoptosis was detected after treatment with quinotrierixin (0.1 μM to 1 μM) for 24 h (Figure 2A). In contrast, apoptotic cells were observed in ARPE-19 cells treated with hydroquinone, a potent prooxidant that as a positive control (Figure 2B).

### Quinotrierixin inhibited X-box binding protein 1 splicing and endoplasmic reticulum stress response in ARPE-19 cells

**Figure 3:**
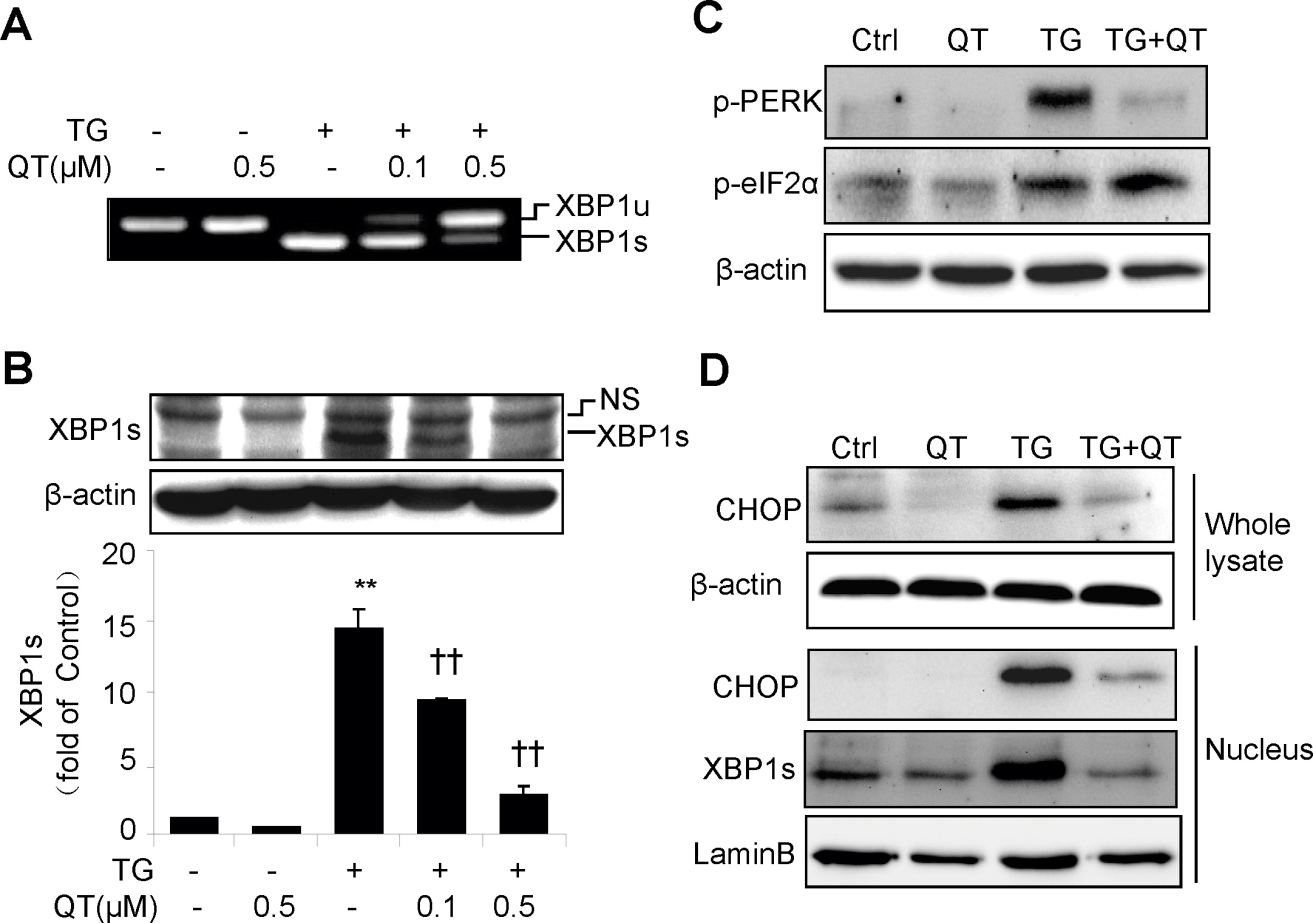
Quinotrierixin inhibits X-box binding protein 1 splicing and C/EBP homologous protein expression in retinal pigment epithelium cells during endoplasmic reticulum stress. ARPE-19 cells were treated with endoplasmic reticulum stress inducer thapsigargin (TG) with or without quinotrierixin for 8 h. **A**: X-box binding protein 1 (XBP1) mRNA splicing was detected with reverse transcription polymerase chain reaction (RT–PCR). XBP1u: unspliced XBP1. XBP1s: spliced XBP1. **B**: Protein level of spliced XBP1 was detected with western blot analysis. NS: none specific. Quantitative data are expressed as mean±SD (n=3 independent experiments). **p<0.01 versus control, ††p<0.01 versus TG. **C**: Phosphorylation of PKR-like endoplasmic reticulum kinase (PERK) and eukaryotic translation initiation factor 2α (eIF2α) was detected with western blot analysis. **D**: Intracellular level of C/EBP homologous protein (CHOP) and nuclear levels of spliced XBP1 and CHOP were determined with western blot analysis. Lamin B was used as loading control for nuclear proteins. The concentrations of TG and quinotrierixin were both 0.5 μM unless otherwise indicated.

Thapsigargin (TG) is a commonly used ER stress inducer that inhibits the ER localized Ca^2+^-dependent ATPase [23]. To determine the effect of quinotrierixin on ER stress response in RPE cells, we incubated ARPE-19 cells with TG in the presence or absence of quinotrierixin and examined ER stress response including XBP1 splicing and phosphorylation of PERK. The results show that quinotrierixin dose-dependently inhibited TG-induced XBP1 mRNA splicing as assessed with RT–PCR in Figure 3A. Consistently, quinotrierixin reduced the protein content of the spliced XBP1 in ARPE-19 cells (Figure 3B). In addition, quinotririxin suppressed TG-induced PERK phosphorylation, but paradoxically enhanced phosphorylation of eIF2α, a downstream substrate of PERK (Figure 3C). Furthermore, quinotrierixin abrogated TG-induced production of CHOP, a major proapoptotic transcription factor that mediates ER stress-driven apoptosis. Moreover, we confirmed that nuclear levels of XBP1 and CHOP, both of which were transcription factors induced during ER stress, were markedly reduced by quinotrierixin in TG-treated cells (Figure 3D).

### Overexpression of spliced X-box binding protein 1 partially reversed the inhibition of retinal pigment epithelium cell proliferation by quinotrierixin

**Figure 4:**
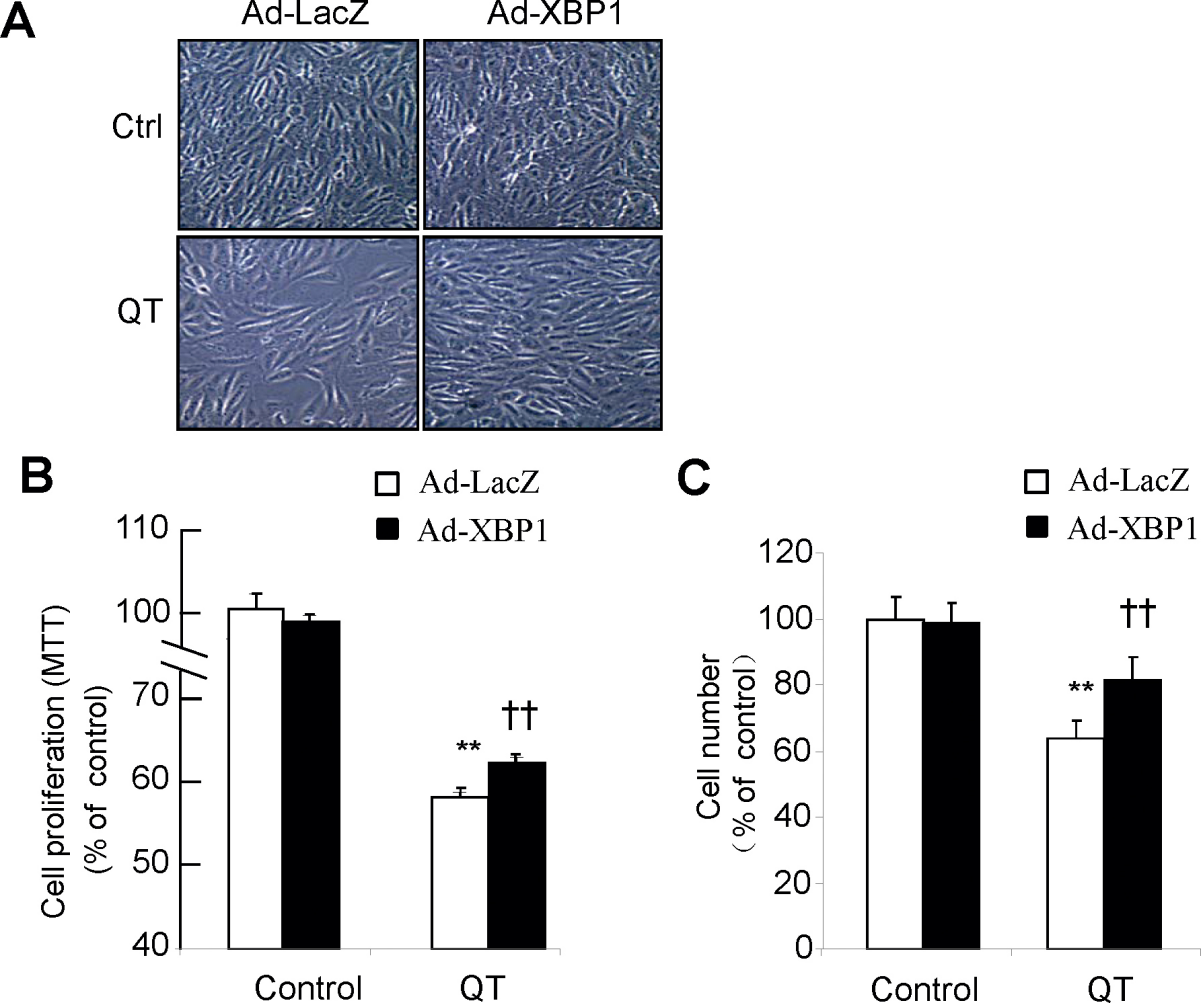
Overexpression of spliced X-box binding protein 1 partially reverses the inhibition of retinal pigment epithelium cell proliferation by quinotrierixin. ARPE-19 cells were transducted with adenovirus expressing spliced XBP1 or LacZ as control and then treated with quinotrierixin 0.5 μM for 24 h. **A**: Phase contrast images are presented. **B**-**C**: Cell proliferation was determined with MTT (**B**) or cell counting (**C**). Quantitative data are expressed as mean±SD (n=4 in each group). **p<0.01 versus control, ††p<0.01 versus QT.

XBP1 is an important transcription factor implicated in cell survival, proliferation, ER stress response, lipid metabolism, and immune cell function, and only the spliced form of XBP1 is an active transcription factor [24]. To investigate whether a reduced level of spliced XBP1 contributes to inhibiting cell proliferation by quinotrierixin, we overexpressed spliced XBP1 in ARPE-19 cells with adenoviral transduction. Overexpressing spliced XBP1 in ARPE-19 cells did not cause changes in cell morphology (Figure 4A), or cell viability as assessed with MTT assay, or cell counting (Figure 4B,C). However, overexpressing spliced XBP1 in ARPE-19 cells partially reversed the inhibition of RPE cell proliferation by quinotrierixin (Figure 4A-C).

## Discussion

In this manuscript, we report that quinotrierixin inhibits cell proliferation without affecting cell viability in cultured human RPE cells. Quinotrierixin was initially identified by Kawamura and colleagues in 2008 while screening inhibitors for ER stress-induced XBP1 activation [16]. As a novel triene-ansamycin group compound, quinotrierixin dose-dependently inhibits XBP1 mRNA splicing with an IC_50_ of 0.082 μM in HeLa cells. In addition, quinotrierixin inhibits tumor cell growth, and the inhibitory effects of quinotrierixin and other triene-ansamycin group compounds on tumor cell growth are highly correlated with their effects against XBP1 activation in tumor cells. This suggests that quinotrierixin may suppress tumor cell growth by inhibiting XBP1. Indeed, overexpression of XBP1 increases proliferation in breast cancer cells and prevents antiestrogen therapy-induced cell cycle arrest [25]. In contrast, knockdown of XBP1 with small interfering RNA effectively slows down the proliferation of human prostate epithelial cells (DU145), a mechanism perhaps that XBP1 promotes proliferation through regulating cell cycle protein cyclin A [26]. In the present study, we show that quinotrierixin dose-dependently reduces RPE cell proliferation and overexpression of spliced XBP1 partially reverses the inhibition of cell proliferation by quinotrierixin. These results suggest that inhibiting XBP1 splicing contributes, at least in part, to the inhibitory effect of quinotrierixin on RPE cell proliferation. However, other mechanisms are involved as well. We speculate that one potential mechanism is through inhibition of protein translation [18]. In RPE cells exposed to ER stress, quinotrierixin promotes eIF2α phosphorylation, which slows down protein translation to reduce ER stress (Figure 3). In a recent study, Yamamoto and associates demonstrated that quinotrierixin inhibited protein synthesis with an IC_50_ of 120 nM in HeLa cells; however, the agent inhibited XBP1 splicing at a lower IC_50_ of 85 nM [18]. We found that quinotrierixin inhibits RPE cell proliferation at 0.1 μM and 0.5 μM doses, and at the same doses suppresses XBP1 splicing. This result may suggest that inhibiting XBP1 splicing and protein synthesis contributes additively or synergistically to the effects on cell proliferation in RPE cells.

Another interesting finding from our study is that quinotrierixin does not cause apoptosis despite the agent’s inhibitory effect on cell proliferation in human RPE cells. As shown in our recent study, XBP1 is an important survival factor for RPE cells, and genetic inhibition of XBP1 by small interfering RNA resulted in apoptosis in ARPE-19 cells [27]. However, apoptosis is not observed in the cells treated with quinotrierixin from 0.1 μM to 1 μM, at which doses quinotrierixin is sufficient to inhibit XBP1 splicing. To explore the potential mechanism, we looked at other ER stress signaling components including the proapoptotic transcription factor CHOP, a prominent mediator of ER stress-driven apoptosis. We found that the intracellular and nuclear levels of CHOP were markedly decreased in cells treated with quinotrierixin. This may in part explain the observation that quinotrierixin does not induce apoptosis in RPE cells but significantly reduces cell proliferation. Nevertheless, the results obtained from the present study, i.e., quinotrierixin inhibits RPE cell proliferation without inducing apoptosis, indicate that quinotrierixin may be used as a relatively safe antiproliferation drug for treating PVR. Future studies should investigate the in vivo effect of quinotrierixin on RPE proliferation and the potential effect on photoreceptors and retinal function in animal models of PVR.
